# Evolution of Precision Medicine and Surgical Strategies for Bicuspid Aortic Valve-Associated Aortopathy

**DOI:** 10.3389/fphys.2017.00475

**Published:** 2017-07-10

**Authors:** Ali Fatehi Hassanabad, Alex J. Barker, David Guzzardi, Michael Markl, Chris Malaisrie, Patrick M. McCarthy, Paul W. M. Fedak

**Affiliations:** ^1^Section of Cardiac Surgery, Department of Cardiac Sciences, Cumming School of Medicine, Libin Cardiovascular Institute of Alberta, University of CalgaryCalgary, AB, Canada; ^2^Department of Radiology, Feinberg School of Medicine, Northwestern UniversityChicago, IL, United States; ^3^Department of Bioengineering, Feinberg School of Medicine, Northwestern UniversityChicago, IL, United States; ^4^Martha and Richard Melman Family Bicuspid Aortic Valve Program, Division of Cardiothoracic Surgery, Bluhm Cardiovascular Institute, Northwestern UniversityChicago, IL, United States

**Keywords:** bicuspid aortic valve, aortopathy, MRI, biomarkers discovery, precision medicine

## Abstract

Bicuspid aortic valve (BAV) is a common congenital cardiac malformation affecting 1–2% of people. BAV results from fusion of two adjacent aortic valve cusps, and is associated with dilatation of the aorta, known as bicuspid valve associated aortopathy. Bicuspid valve aortopathy is progressive and associated with catastrophic clinical events, such as aortic dissection and rupture. Therefore, frequent monitoring and early intervention with prophylactic surgical resection of the proximal aorta is often recommended. However, the specific pattern of aortopathy is highly variable among patients, with different segments of the ascending aorta being affected. Individual patient risks are sometimes difficult to predict. Resection strategies are informed by current surgical guidelines which are primarily based on aortic size and growth criteria. These criteria may not optimally reflect the risk of important aortic events. To address these issues in the care of patients with bicuspid valve aortopathy, our translational research group has focused on validating use of novel imaging techniques to establish non-invasive hemodynamic biomarkers for risk-stratifying BAV patients. In this article, we review recent efforts, successes, and ongoing challenges in the development of more precise and individualized surgical approaches for patients with bicuspid aortic valves and associated aortic disease.

## Introduction

Bicuspid aortic valve (BAV) is the most common congenital heart defect, affecting 1–2% of the general population (Hoffman and Kaplan, 2002). Abnormality of the aorta is frequently associated with BAV, with thoracic aortic dilation seen in approximately 40% of patients in referral centers (Masri et al., 2016). Consequently, compared to the general population, patients with BAV are at a higher risk for acute aortic emergencies, such as aortic dissection (Januzzi et al., 2004). Given the high morbidity and mortality associated with these emergencies, identifying the optimal timing to intervene, and prevent such events is of paramount importance. However, this is a challenging process as many factors, including patient age, comorbidities, presence or absence of aortic valvular disorders, and family history of BAV, could all affect management.

Over the past three decades, it was perceived that aortopathy associated with BAV, “bicuspid aortopathy,” had a similar pathophysiology to aortic disorders associated with tricuspid aortic valve (TAV) disease. Specifically, it was believed that turbulent or eccentric flow resulting from a narrowed orifice (BAV) led to aortic dilation. However, several ensuing studies demonstrated a strong genetic component for BAV-associated aortopathy in this patient population, which in turn, significantly increases the risk of acute aortic events. These initial findings led to recommendations for more aggressive management approaches, which viewed bicuspid aortopathy in the same light as Marfan's syndrome, thereby advocating for earlier surgical intervention for patients with BAV disease. More recent research, however, has implied that genetic predisposition and hemodynamic irregularities contribute to varying degrees in different subgroups of BAV patients, and the rate of aortic complications is not as high as previously believed (Fedak et al., 2005; Hiratzka et al., 2010; Girdauskas et al., 2011; Itagaki et al., 2015; Sherrah et al., 2016). These recent studies emphasize the importance of identifying the underlying cause of bicuspid aortopathy as it has different therapeutic implications for patients with or without BAV presenting with aortic pathologies.

A few groups have considered the optimal management of BAV-associated aortopathy, and several documents have addressed it, with the first being a multi-societal set of guidelines published in 2010 (Hiratzka et al., 2010). In the more recently published guidelines by the American Heart Association/American College of Cardiology (AHA/ACC) on valvular heart disease, a more conservative set of recommendations were made (Nishimura et al., 2014). Given the significant difference in recommendations, a recent clarification statement was published (Hiratzka et al., 2016). The European Society of Cardiology has also made more conservative recommendations for the management of bicuspid aortopathy (Vahanian et al., 2012; Erbel et al., 2014). In addition, the American Association for Thoracic Surgery (AATS) will be releasing an expert consensus statement in 2017.

Emerging research is considering the genetics and molecular and cellular mechanisms underlying the disease. As Prakash and colleagues elegantly outline, autosomal-dominant transmission of BAV was observed in some 3-generation pedigrees, but there is no single-gene model which clearly explains BAV inheritance. The prevalence of BAV stands nearly 10-fold higher in primary relatives of patients with BAV than in the general population, further supporting the notion that genetics does indeed play an important role (Prakash et al., 2014). To better understand the mechanisms which drive BAV and bicuspid aortopathy, different groups are studying various molecular pathways and genetic foci. Thus far, NOTCH1 remains the only gene which has been implicated for isolated BAV identified using linkage analysis and positional cloning strategies, despite probably being the cause of small proportion of familial cases (Garg et al., 2005; Ellison et al., 2007). These studies are all in their infancy, but continued basic research in this area will undoubtedly shed more light onto the genetic building block of BAV and bicuspid aortopathy.

Bicuspid aortopathy is a very heterogeneous disorder, a feature which has added to the complexity of devising management guidelines. For example, in some instances, despite developing aortopathy, patients can be asymptomatic throughout their life. Moreover, dilation of the aorta may occur in the aortic root, ascending aorta, proximal aortic arch, or a combination of any of these three (Fazel et al., 2008). Moreover, despite ongoing research, it remains to be established if medical therapy is effective in preventing complications for patients with bicuspid aortopathy. Although supportive clinical evidence is still missing, beta-blockers and angiotensin receptor blocking agents are frequently prescribed to protect the BAV-aorta within this patient population (Danyi et al., 2011; Chun et al., 2013; Ziganshin et al., 2015). On the other hand, several groups have studied the risk of developing aneurysmal dilation of the ascending aorta over time in patients with BAV (to a size of 4.0–4.5 cm). It was shown that 20–30% of patients with BAV develop aneurysmal enlargement during 9–25 years of follow up (Michelena et al., 2008, 2014; Tzemos et al., 2008). In fact, in a recent review paper, based on eight independent studies, it was suggested that up to 84% of patients with BAV ultimately develop an aneurysm, and the risk of the aneurysm development was 80-fold higher when compared to the general population (Michelena et al., 2014; Wasfy et al., 2015).

Of clinical significance, dilatation of any or all segments of the aorta is seen in approximately 50% of patients with BAV (Fedak et al., 2005), and ascending aortic aneurysms occur in 1% of BAV patients per year. Although bicuspid aortopathy can manifest in all segments of the aorta, it is more often isolated to the aortic root, ascending aorta, or proximal aortic arch. Most patients will present with maximal dilatation of the tubular mid-ascending aorta, specifically at the greater curvature, with the aortic root and proximal arch being affected to varying degrees. Thus, resection strategies can vary greatly (Fedak et al., 2005; Della Corte et al., 2014a; Fedak and Verma, 2014; Adamo and Braverman, 2015; Moon, 2015; Sundt, 2015). In addition to deciding when to intervene in replacing the aorta in bicuspid aortopathy, assessing what to resect also poses a clinical dilemma (Fedak and Verma, 2014; Sundt, 2015). Bicuspid aortopathy is progressive, increasing the risk of aortic dissection and rupture. To date, these complications have been challenging to predict. Therefore, frequent monitoring and personalized interventions for both timing of surgery and the extent of resection are of paramount importance in preventing these clinical catastrophes and delivering optimal care (Itagaki et al., 2016).

Although different international societies and expert groups have provided unified guidelines regarding optimal management of patients with BAV disease, most surgical recommendations have primarily been based on maximal aortic diameter and growth rate (Nishimura et al., 2014). According to these guidelines, prophylactic replacement of the ascending aorta is performed in roughly 25% of BAV patients within 25 years from the time of diagnosis (Michelena et al., 2011). This has significant implications, as the burden of surgery for BAV patients in the United States exceeds 1 billion dollars per year, and surgical intervention has doubled over the past decade (Opotowsky et al., 2013). It is noted that surgical planning and decision-making for BAV patients is affected by physician bias and historical local practice within institutions, which aren't always consistent and in line with guidelines (Verma et al., 2013; Della Corte et al., 2014b; Girdauskas and Borger, 2014; Michelena et al., 2014; Verma and Siu, 2014; Sundt, 2015; Wasfy et al., 2015). In a recent survey of 100 cardiac surgeons, it was postulated that attitudes on the etiology, inherited aortopathy vs. acquired from hemodynamic stress, rather than proven clinical evidence dictated surgical treatment of BAV aortopathy (Verma et al., 2013). Undeniably, this has complicated widely accepted and universally utilized guidelines, emphasizing the need for more translational and clinical research solely dedicated to BAV patient populations.

Fortunately, over the past 3 years, a concerted effort has been made in understanding the individual variability inherent to BAV disease and the role hemodynamic factors play in its manifestation and progression (Della Corte et al., 2014a; Fedak and Verma, 2014; Girdauskas and Borger, 2014; Martin et al., 2014; Uretsky and Gillam, 2014; Verma and Siu, 2014; Michelena, 2015; Spinale and Bolger, 2015; Itagaki et al., 2016; Sievers et al., 2016). There is a general consensus among experts regarding a critical need in developing personalized risk assessments beyond conventional aortic size and growth criteria, in delivering optimal care to BAV patients. The challenge clinicians face today is a paucity of prognostic models to inform the timing and extent of surgical intervention. To address some of these issues, our translational research group and others have focused on validating use of novel imaging techniques to establish non-invasive hemodynamic biomarkers for risk-stratifying BAV patients. In this review article, we will consider recent efforts, successes, and ongoing challenges in the development of more precise and individualized surgical approaches for patients with BAVs and associated aortic disease.

## Pathophysiology and wavering guidelines

**Figure 1:**
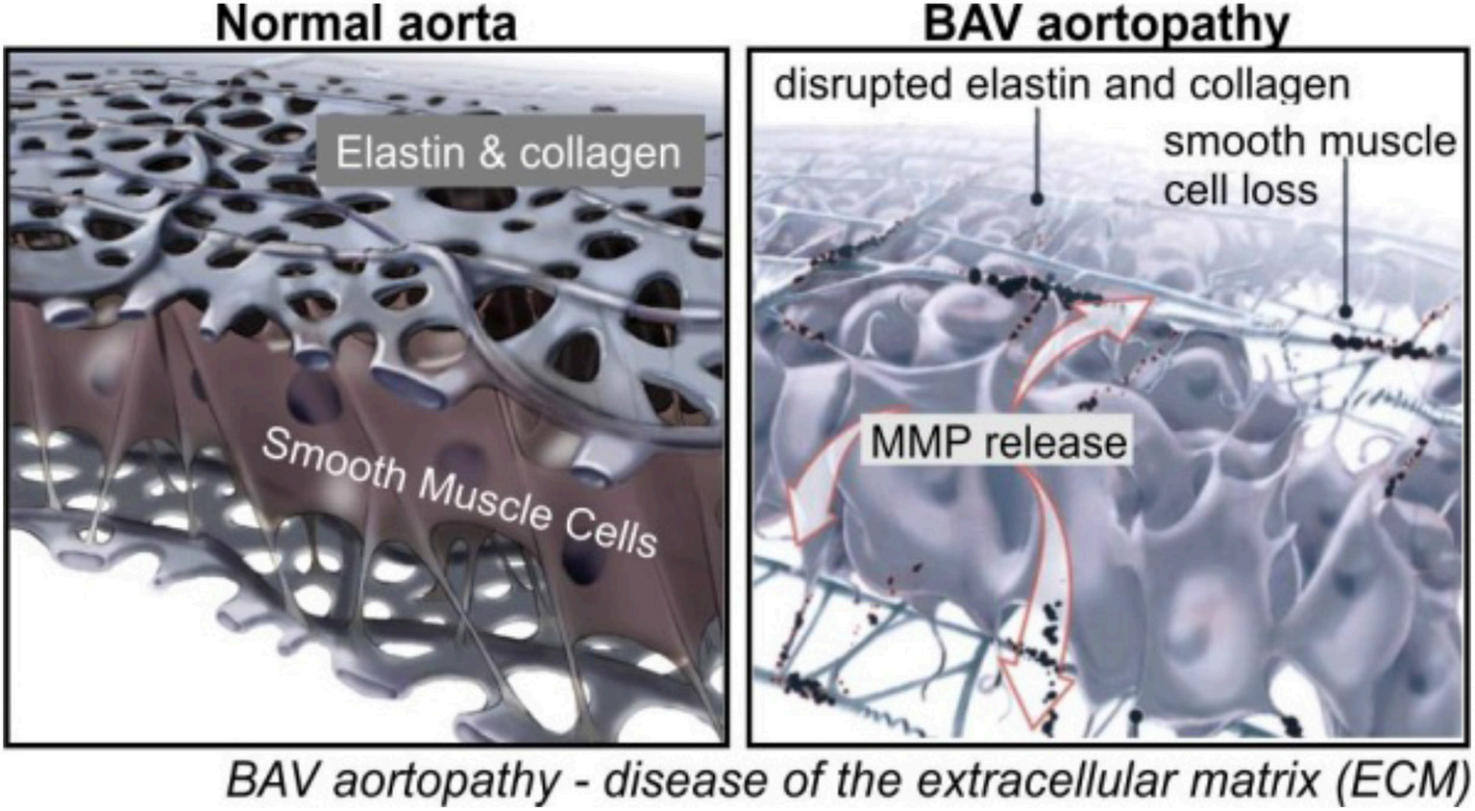
Aortic wall degeneration in BAV (Fedak et al., 2002).

To develop patient-specific parameters in BAV populations, researchers have considered the pathophysiology of BAV aortopathy. Like other vessels, a normal aortic wall is divided into three layers: intima, media, and adventitia. Elastin fibers, vascular smooth muscles cells, and structural extracellular matrix (ECM) comprise the medial layer. The aortic media regulates tissue biology and biomechanics (Figure 1). Different studies have demonstrated that BAV occurs in conjunction with degeneration of this layer. Bicuspid aortopathy involves medial ECM abnormalities which include (Della Corte et al., 2014b; Itagaki et al., 2016)

1. ECM dysregulation: altered matrix metalloproteinase expression and activity (Thompson and Cockerill, 2006; Ikonomidis et al., 2007, 2012; Wilton et al., 2008; Fedak et al., 2013; Rabkin, 2014)
2. Altered medial ECM architecture: elastin fiber degeneration (de Sa et al., 1999; Bauer et al., 2002; Cotrufo et al., 2003, 2005; Chung et al., 2007; Phillippi et al., 2014)
3. Tissue dysfunction: altered stiffness and biomechanics (Nistri et al., 2002, 2008; Schaefer et al., 2007; Pees and Michel-Behnke, 2012; Oulego-Erroz et al., 2013; Warner et al., 2013; Forsell et al., 2014; Moaref et al., 2014; Petrini et al., 2014)

As expected, the presence, severity, and location of these pathologies differs among patients. This poses a significant question: in addition to a possible genetic predisposition to dilatation, do hemodynamic conditions in the aorta contribute to its remodeling in BAV patients?

**Figure 2:**
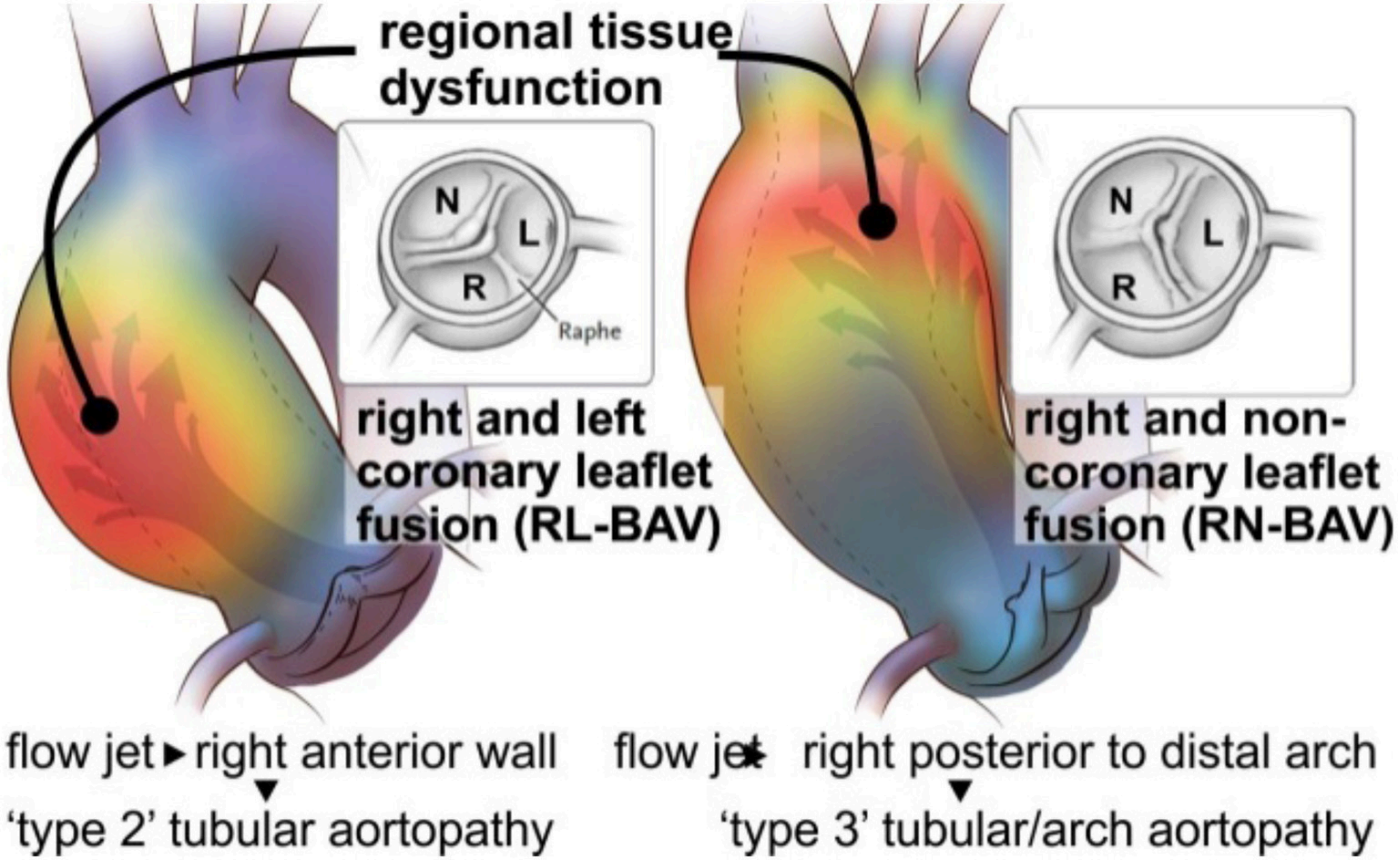
Valve mediated hemodynamics. BAV fusion patterns lay the foundation for changes in aortic outflow and wall shear stress (WSS). Eccentric blood flow from the RL-BAV impinges on regions of dilatation at the tubular ascending aorta wall. Flow from the RN-BAV reflects off the proximal posterior wall and impinges on regions of aortic dilatation within the proximal arch. Adapted with permission (Itagaki et al., 2016).

Unfortunately, the mechanisms which contribute to aortopathy in BAV patients have not been clearly elucidated (Davies et al., 2007; Tadros et al., 2009; Girdauskas et al., 2011; Michelena et al., 2011; Sievers and Sievers, 2011), and it is not known whether genetics leads to aortopathy or if the altered BAV morphology results in isolated diseased areas within the aortic wall secondary to abnormal blood flow from the valve (Figure 2). It is possible that it is a combination of both factors, but a unilateral focus on the genetic component has supported aggressive surgical intervention with respect to the timing and extent of aortic resection. Although previous guidelines and size thresholds for surgical resection were based on algorithms similar to those for patients diagnosed with genetic aortopathies, such as Marfan's syndrome (Bonow et al., 2008), recent clinical data strongly suggest that bicuspid aortopathy is distinct from that of Marfan's (Itagaki et al., 2015). Not surprisingly, clinical approaches in managing BAV aortopathies are highly influenced by different opinions on the varied impact of genetics and hemodynamics on disease progression (Hardikar and Marwick, 2015). Initially, a conservative cut off of 5.5 cm was used in 1998 (Tricoci et al., 2009). In 2010, surgeons were more aggressive, intervening when the aortic diameter was 4.0–4.5 cm (Warnes et al., 2008), but reverted to a more conservative approach of 5.5 cm cut off in 2014 (Svensson et al., 2013; Erbel et al., 2014; Michelena et al., 2015). Throughout this time no clinically and scientifically proven study was reported to support either a conservative or aggressive approach in surgical resection in bicuspid aortopathy (Hardikar and Marwick, 2015). To offer scientifically proven guidelines, which would consistently be used by clinicians, it is of paramount importance to continue the work on the discovery and implementation of novel aortic risk markers (Michelena et al., 2015; Spinale and Bolger, 2015).

## New evidence on the pathophysiology of BAV aortopathy

Previous work studying the hemodynamic component to BAV-related aortopathy focused on the severity of aortic valve stenosis (AS) or insufficiency (AI) (Tzemos et al., 2008; Girdauskas et al., 2011; Michelena, 2015). It is now believed that these conventional hemodynamic factors alone do not reflect the impact on the aortic wall due to a malformed valve (Girdauskas et al., 2011; Sievers and Sievers, 2011; Atkins and Sucosky, 2014; Della Corte et al., 2014b; Adamo and Braverman, 2015; Michelena et al., 2015; Moon, 2015). These findings imply that the development of bicuspid aortopathy is not primarily driven by a genetic predisposition. Further supporting these results are recent studies by our group and others which have shown that altered aortic flow and valve morphology in BAV patients are related to the expression of the aortopathy phenotype (Kang et al., 2013; Mahadevia et al., 2014; Prakash et al., 2015). As depicted in Figure 2, 4-D flow MRI studies provide strong evidence that valve-mediated local flow dynamics (Barker et al., 2012) and regional differences in wall shear stress (WSS) (Mahadevia et al., 2014) are associated with changes in regional aortic wall histology and proteolytic events (Guzzardi et al., 2015), contributing to unfavorable aortic remodeling. More significantly, these preliminary data can be landmark findings in better understanding valve-mediated hemodynamics' impact on the progression of bicuspid aortopathy. They can also be a platform for clinically-proven justification in utilizing MRI-based biomarkers in risk stratification.

The dearth of prognostic models to assist in the surgical management of BAV patients is the biggest challenge clinicians face today, particularly with regards to the timing and extent of surgical repair. As mentioned, this patient population is often offered aortic resection primarily based on maximal aortic size dimension and the rate at which the aorta expands. It is, however, now understood that measures of aortic size alone are insufficient to dictate treatment algorithms (Della Corte et al., 2012a; Verma et al., 2013; Della Corte, 2014; Michelena et al., 2014; Prakash et al., 2015; Sundt, 2015; Wasfy et al., 2015). Therefore, substantial efforts are currently being made to improve risk prediction for aortic catastrophes in BAV patients (Della Corte et al., 2007, 2012b,c, 2013; Ikonomidis et al., 2013).

Recent work has yielded strong evidence that including measures of downstream valve-mediated hemodynamics into the work-up of BAV patients has a high likelihood to circumvent current prognostic challenges (Davies et al., 2007; Rabkin, 2014; Adamo and Braverman, 2015; Song, 2015). However, conventional diagnostic modalities, such as Doppler echocardiography, 2D phase contrast [PC]-MRI, and CT scan, do not offer the means for a thorough *in-vivo* assessment of three dimensional blood flow through the aorta, which is necessary to study the role of transvalvular hemodynamics on the downstream forces experienced at the aortic wall and their impact on the progression of aortic dilatation.

## Innovative technology—4D flow MRI

**Figure 3:**
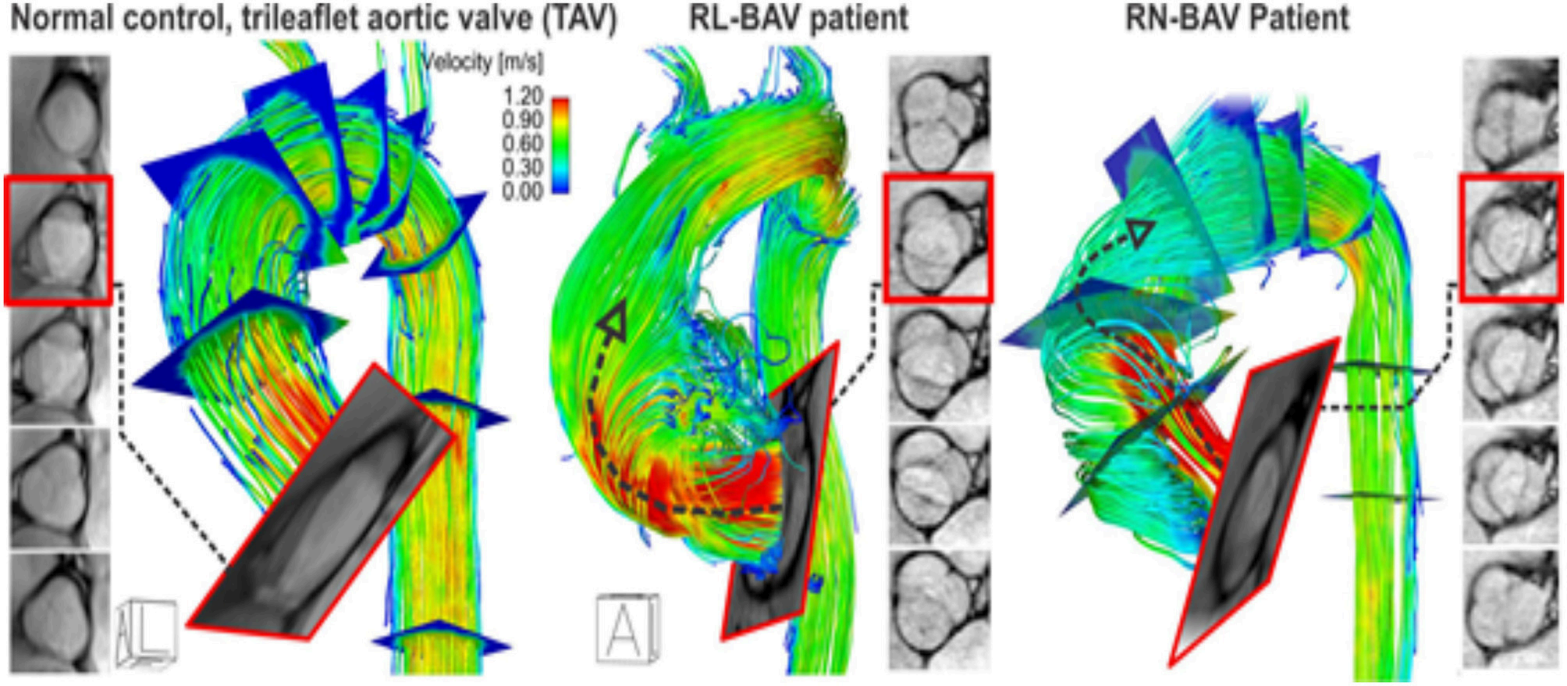
4D flow MRI in a control and BAV patient with a right-left (RL) fusion pattern and right-non-coronary (RN) fusion pattern. Note that the RL-BAV exerted significant eccentric aortic outflow jet (but not higher velocity, arrow) compared to TAV. The BAV phenotype (RL vs. RN) greatly influences aortic outflow, which in turn, affects aortic segments exposed to elevated WSS (Figure 4). Adapted with permission (Barker et al., 2012; Guzzardi et al., 2015).

Recent advances in magnetic resonance imaging have allowed for uncompromised *in-vivo* assessment of time-resolved 3D blood velocity, using a volumetric technique, referred to as 4D flow MRI (Figure 3). This modality provides an opportunity to quantify complex three dimensional blood flow patterns *in-vivo*, and has facilitated new insights into sophisticated cardiovascular hemodynamics (Harloff et al., 2009, 2010b; Markl et al., 2010, 2011a; Barker et al., 2012; Mahadevia et al., 2014). In particular, multidimensional 4D flow MRI data, which infers three spatial dimensions describing 3D velocity over time, permits visualization of aortic blood flow, quantification of regional velocity and flow (Markl et al., 2007, 2011b; Frydrychowicz et al., 2008a; Bock et al., 2010), and WSS (Stalder et al., 2008; Barker et al., 2010; Harloff et al., 2010a; Bock et al., 2011; Garcia et al., 2014; van Ooij et al., 2015a).

**Figure 4:**
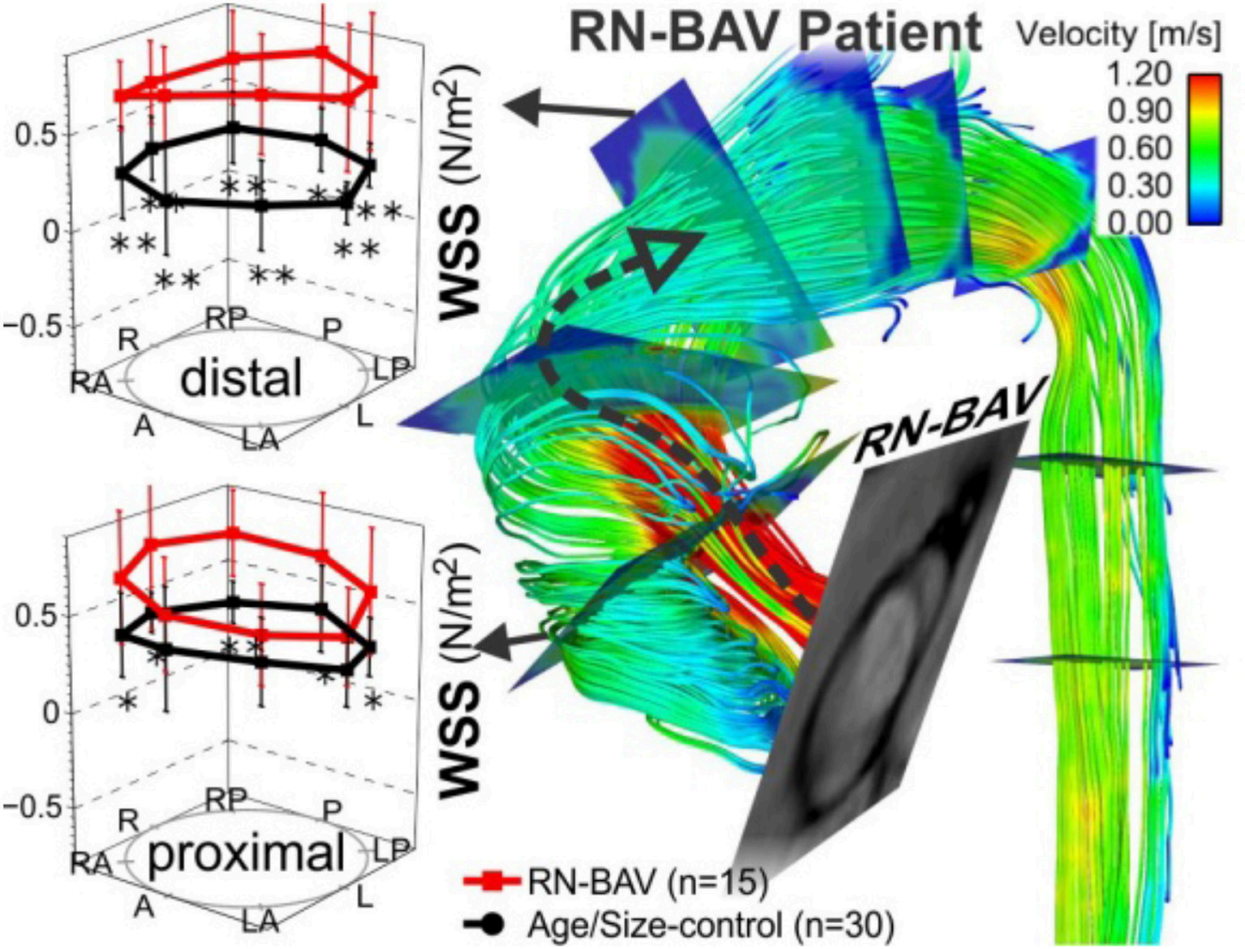
4D flow & WSS quantification in 15 RN-BAV patients found that 4D flow can detect significant differences (^*^p < 0.05, ^**^p < 0.001) in aortic WSS distribution compared to 30 TAV controls. **(A)** Control populations and **(B)** individual BAV patient. Note the different aortic outflow pattern compared to the RL-BAV patient in Figure 3 (Barker et al., 2012).

Multiple institutions have now shown that 4D flow MRI can be utilized to accurately identify altered flow patterns secondary to BAV, even if aortic stenosis is present (Figures 3, 4). Among the observed hemodynamic changes are eccentric flow patterns, which result in a change of the drag forces at the vessel wall (Figure 4). Despite what is believed to be a multifactorial disease, recent studies have shown WSS to play a major role in bicuspid aortopathy. We now believe that WSW may change local matrix homeostasis, and consequently affect the structure of the ascending aorta (Stalder et al., 2008; Markl et al., 2009; Harloff et al., 2010a; Markl et al., 2010; Bock et al., 2011; Garcia et al., 2014; van Ooij et al., 2015a). In fact, research has shown WSS to affect cell function, implicating its role in the development of aortopathy (den Reijer et al., 2010; Hope et al., 2010a, 2011).

Our group has used non-invasive MRI techniques (2D phase contrast MRI) to show BAV-mediated alterations in flow and WSS (Barker et al., 2010). Building on the successes of our initial work, we then employed 4D flow MRI to definitively demonstrate aortic WSS was increased in BAV subjects independent of the degree of stenosis when compared to age and aortic size-matched controls (*P* < 0.05, Figure 4; Mahadevia et al., 2014). Also of clinical significance, we have shown that regional variation of WSS with the aorta is dependent on aortic valve fusion phenotype (Barker et al., 2012; Mahadevia et al., 2014), and is associated with the diameter of the aorta (Bissell et al., 2013). In one of our recent studies, we considered 30 BAV patients and 30 age-appropriate trileaflet aortic valve (TAV) controls, and showed that altered aortic hemodynamics may be a mechanism by which right and left coronary leaflet (RL-BAV) or right and non-coronary leaflet valve (RN-BAV) fusion influences the expression of aortopathy (Mahadevia et al., 2014).

A significant finding has been the fact that hemodynamic alterations are related to medial wall degeneration (Guzzardi et al., 2015). A pilot study was recently completed, which included both *in-vivo* 4D flow MRI and aortic tissue resection in 20 BAV patients. The study successfully demonstrated the ability to correlate *in-vivo* 4D flow derived hemodynamic biomarkers with tissue metrics of bicuspid aortopathy. In this work, 20 BAV patients undergoing aortic resection underwent pre-operative 4D flow MRI to regionally map WSS and had histologic examination of their resected tissue samples. Samples obtained from regions of both elevated and normal WSS within the same patient were paired, and compared for medial elastin degeneration by histology and ECM dysregulation by protein expression. As depicted in Figures **6**, 7, regions of increased WSS showed greater medial elastin degradation compared to adjacent segments with normal WSS. Moreover, multiplex protein analyses of ECM regulatory molecules revealed an increase in TGFβ-1, MMP-1, MMP-2, MMP-3, and TIMP-1 in increased WSS areas, suggesting ECM dysregulation in regions of elevated WSS. In a much larger prospective cohort, the aim will be to more comprehensively characterize aortic tissues resected from segments of abnormal WSS, further clarifying the impact of altered blood flow.

## The promise of personalized medicine for BAV patients

The pilot study demonstrated the potential utility of 4D flow MRI to identify areas with more advanced aortopathy in patients. Future work will focus on these significant findings, with the objectives being the discovery and validation of key hemodynamic imaging biomarkers. This should include refining aortic MRI protocols, creating “maps” or “atlases” of normal age and gender-matched imaging biomarkers of aortic hemodynamics in both BAV and TAV patients, and pushing current boundaries by carrying out a clinico-pathologic correlation study in BAV and TAV patients with aortopathy to establish imaging biomarkers predictive of aortic tissue pathology and dysfunction.

A limitation of 4D flow MRI is the acquisition time needed and its low blood-tissue contrast, which has proven a challenge for its translation to routine clinical use. Ongoing efforts to decrease exam time with the use of accelerated imaging strategies such as radial undersampling, k-t approaches, or compressed sensing are rapidly becoming available for the clinic (Baltes et al., 2005; Lustig et al., 2007; Moftakhar et al., 2007). This should hopefully result in reduced acquisition times for the assessment of valve dynamics and time-resolved aortic 3D geometry. Moreover, these advances should improve 3D segmentation of various parts of the aorta for precise assessment of hemodynamic imaging biomarkers, such as WSS and flow displacement, and amalgamate the analysis of aortic valve morphology (RL-, RN-BAV, etc.), geometry (orifice area), and dynamics (opening angle).

Additionally, the pilot study considered whether aortic WSS, ECM architecture and protein expression, and non-traditional hemodynamic parameters can affect regional aortic tissue function. However, it is still not known which factors associated with valve-mediated hemodynamics are most sensitive in predicting the development and progression of aortopathy. For instance, WSS gradient (WSSG) and oscillatory shear index (OSI) are also known to promote remodeling (Hope et al., 2010a; Bissell et al., 2013). Moreover, previous work studying WSS was based on two-dimensional imaging planes manually placed in the thoracic aorta (Frydrychowicz et al., 2008b, 2009; Stalder et al., 2008; Markl et al., 2010, 2011c, 2013; Harloff et al., 2010a; Hope et al., 2010b; Barker et al., 2012; Burk et al., 2012; Potters et al., 2014), and was hence, limited in calculating imaging biomarkers along the entire length of the aorta. A future goal would be to develop a comprehensive data analysis protocol.

**Figure 5:**
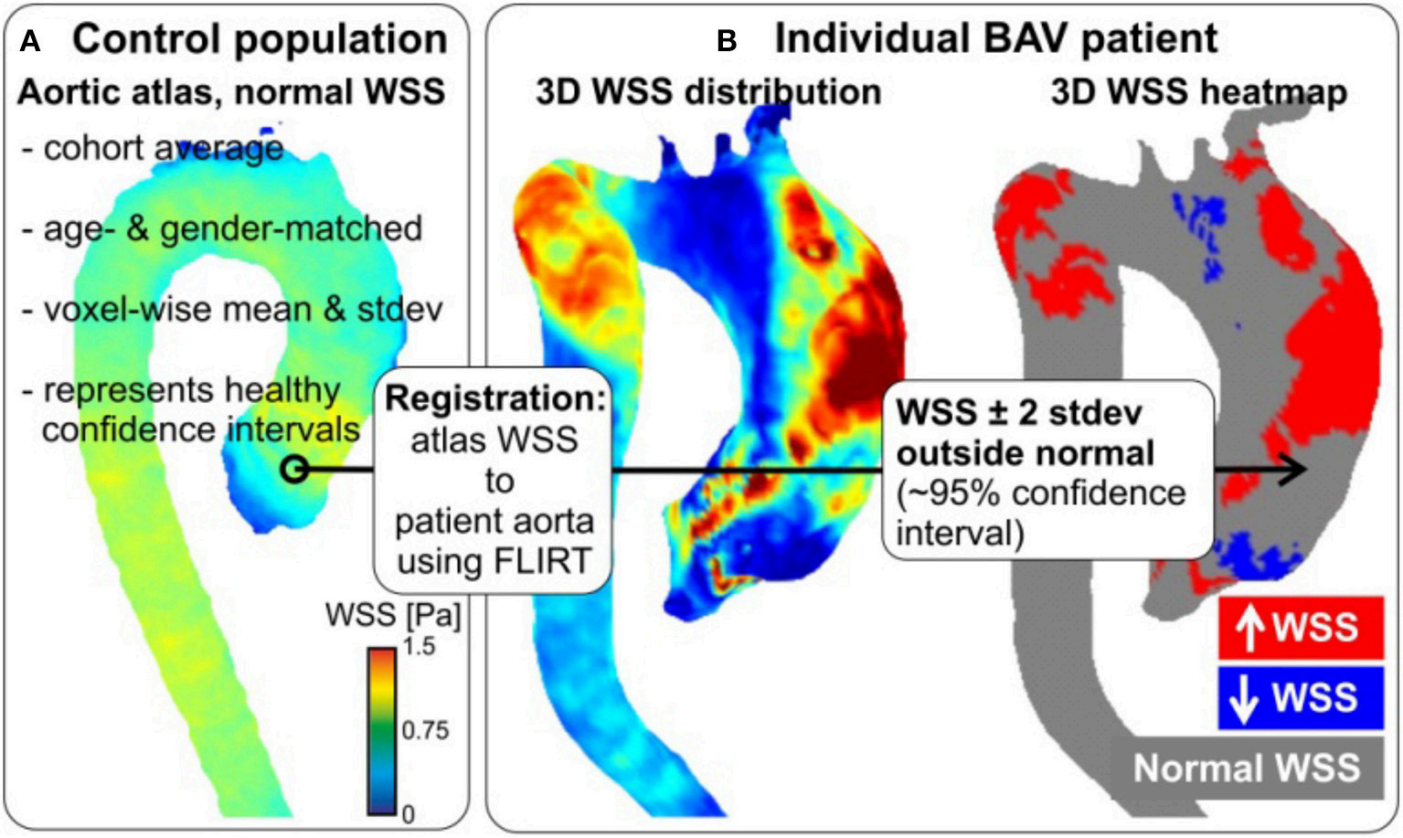
Patient-specific WSS heat maps. Healthy atlases are registered to BAV data with tools developed for brain mapping. Maps can be reliably generated, even in patients with complex aortic geometries (as here).

In a recent study, an algorithm was developed to compute volumetric 3D WSS along the entire surface of the aorta (Figure 5; Potters et al., 2014; van Ooij et al., 2015a). Test–retest experiments for systolic WSS demonstrated excellent accuracy, with a 9% coefficient of variance, and a 6% inter-observer error (van Ooij et al., 2015b). We also now know that elevated WSS could be seen on the outer curvature of the ascending aorta in 13 BAV patients, with a significant correlation to peak systolic velocity (Cibis et al., 2015). Future work will focus on extending the methodology to incorporate additional imaging biomarkers implicated in vessel wall remodeling, such as WSSG and OSI.

Prior research on creating “heat maps” or “atlases” utilized a 3D WSS mapping technique which allowed for compact visualization of hemodynamic parameters studies across multiple subjects. This mapping, however, is limited in that it does not detect where “abnormal” values exist. Therefore, a database of healthy volunteer 4D flow MRI exams was created to produce an aortic “atlas” which established regional confidence intervals for normal physiologic WSS throughout the aorta (Figure 5; van Ooij et al., 2015a). Linear intra- and inter-modal brain image registration (FLIRT, Linear Image Registration Tool, FMRIB, Oxford; Jenkinson and Smith, 2001) were utilized to co-register aortic 3D WSS of 10 TAV patients with no aortic stenosis, but present aortic dilatation, and TAV patients with aortic stenosis, but no aortic dilatation, with the atlas. The dilatation cohort had significantly lower WSS on 7% of the ascending aorta, whereas the stenosis cohort showed significantly higher WSS on 34% of the ascending aorta surface (van Ooij et al., 2015a,c). A future research goal is to build on these efforts to construct age and gender matched atlases of the imaging biomarkers in a large population.

**Figure 6:**
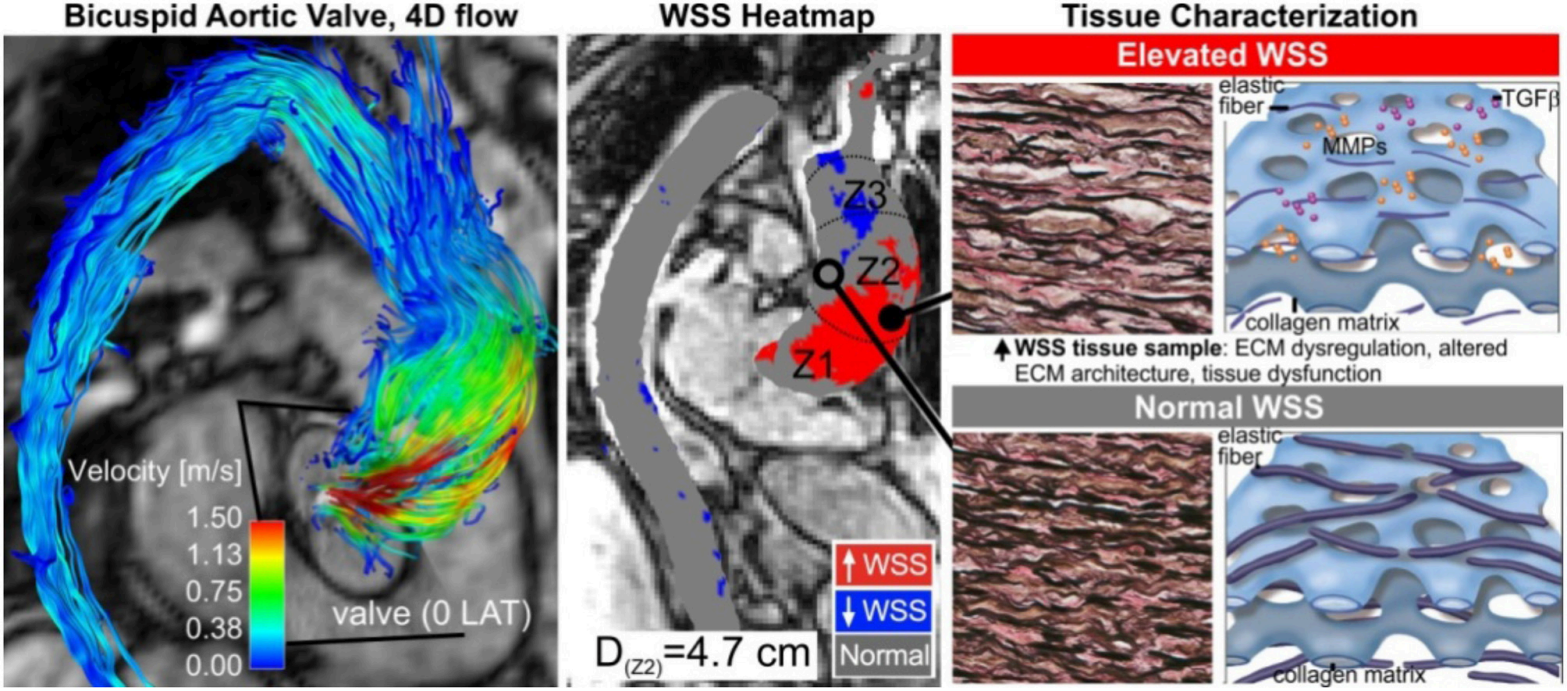
Tissue histopathology. Aortic wall regions are exposed to elevated WSS (middle, red region), due to eccentric transvalvular BAV flow **(left)**. This manifests in the expression of abnormal tissue metrics of aortopathy **(right)**. Adapted with permission (Guzzardi et al., 2015).

In summary, patient-specific WSS can be reliably computed and co-registered to a healthy control atlas, representing the normal ranges of physiologic WSS. These normal WSS atlases can subsequently be employed to create “heat maps” which represent regions of abnormally high or low WSS in a patient in question (Figures 5B, 6; van Ooij et al., 2015c). This heat map provides a foundation for thorough, yet succinct, assessment to detect regions, and segments of the aortic wall which are exposed to abnormal hemodynamics on an individual, patient-specific, basis.

**Figure 7:**
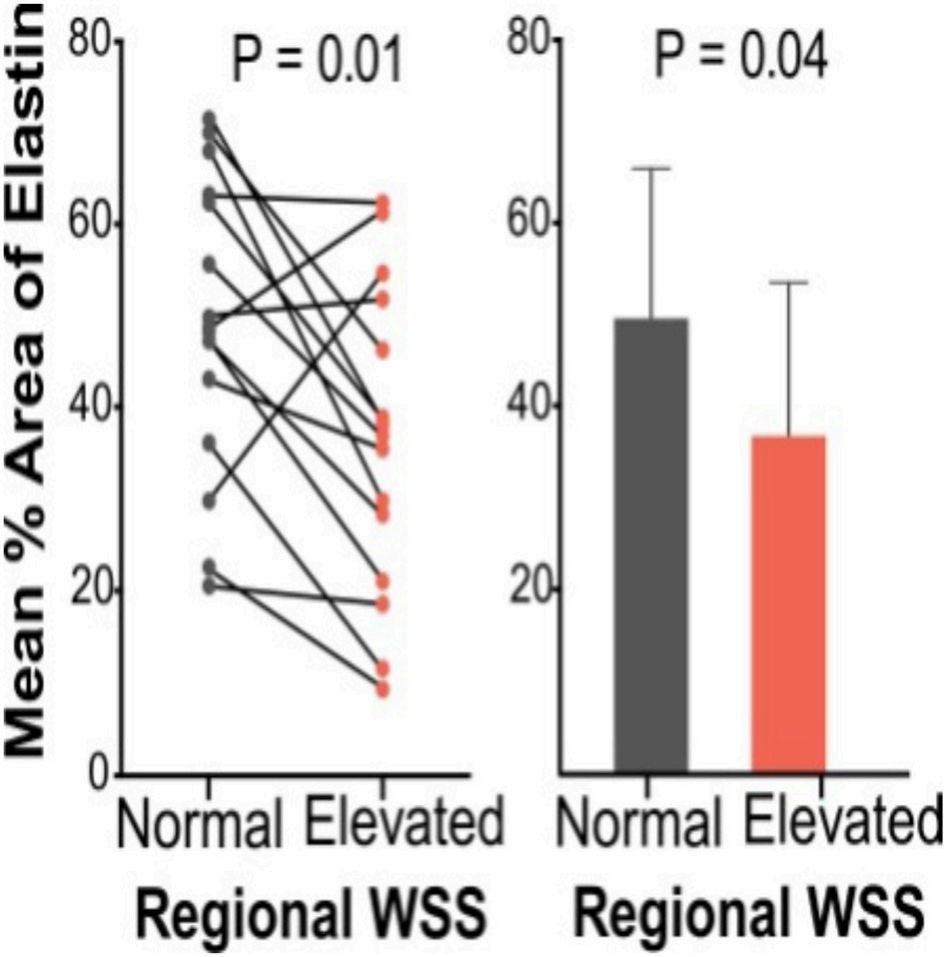
Elastin content from Verhoeff–Van Gieson elastin staining for patient pairs and cumulative group means for aortic wall subjected to normal and elevated WSS (Guzzardi et al., 2015).

## Imaging hemodynamic biomarkers and aortic wall pathology

A major component of personalized medicine is the potential utility of non-invasive biomarkers in diagnosing and prognosticating clinical risk and outcomes. The same holds true for BAV and bicuspid aortopathy; much current investigation is aimed at identifying such markers to improve the management of this patient population. For instance, Della Corte's group recently published a study which shows that the ratio of circulating Transforming Growth Factor Beta-1 to soluble endoglin is an early biomarker for bicuspid aortopathy. Although systemic biomarkers remain to be more fully validated, such investigations provide a foundation for future work in this area (Forte et al., 2017).

The focus of this review, however, is the role of novel non-invasive imaging hemodynamic biomarkers. As mentioned above, to date, most research on the hemodynamic hypothesis for bicuspid aortopathy has focused on aortic valve function, namely the severity of aortic stenosis or insufficiency (Tzemos et al., 2008; Girdauskas et al., 2012; Michelena, 2015). These factors alone, however, do not fully reflect the hemodynamic burden exacted on the aortic wall, secondary to the malformed aortic valve. Moreover, despite not being completely well-understood, there are inherent differences in the development of BAV vs. TAV aortopathy. For instance, the associated aortopathy in most patients with TAV is thought to be a result of aortic valve stenosis and altered post-valve hemodynamics. On the other hand, a strong genetic predisposition has been suggested to contribute to aortopathy in BAV patients. Interestingly, a recent study showed that TAV aortopathy was associated with more severe histologic abnormalities compared to BAV aortopathy, especially when stratified by diameter (Heng et al., 2015). Properly assessing the diagnostic value of novel imaging biomarkers requires further work in this area to gain a better appreciation of the differences in the development of BAV vs. TAV aortopathy.

As alluded to above, recently, a correlation between WSS and regional aortic tissue remodeling in BAV patients was established (Guzzardi et al., 2015). It was concluded that elastin content and structure was significantly disrupted in areas of high WSS with a change in the expression of specific MMPs and TGF-beta. There was also an observed trend toward differences in the elastic modulus and tissue stiffness using biaxial testing. Although further clarification is required, these data promise an opportunity for utilizing valve-mediated hemodynamics as non-invasive biomarkers of aortopathy susceptibility and progression.

Despite making great strides in advancing our understanding of BAV aortopathy and its clinical implications, work has so far considered only one hemodynamic biomarker, systolic WSS, within a small sample size. Future research will need to focus on identifying the hemodynamic metric most predictive of disease severity, as well as elucidating the role other clinical factors play, in a larger population sample size. To determine the differential impact and magnitude of valve-mediated hemodynamics as compared to genetic predisposition and other non-hemodynamic factors in BAV patients, future work should compare our results to a purely hemodynamic-mediated aortopathy reference group. Finally, the pilot study was not powered to assess difference in MMP-2 expression, thus ongoing and future studies can consider MMP-2 activity, histopathology, and biomechanics in larger samples sizes.

## Conclusion

Bicuspid aortic valve is the most common congenital cardiac defect. Multiple studies provide strong evidence for the clinical significance of this disease, especially as how it relates to pathologic abnormalities of the aorta. Many studies have also shown the devastating sequelae of aortic complications in patients with BAVs. All of these impose a highly unfavorable health and economic burden on patients and society-at-large. To better understand the etiology and pathophysiology of bicuspid aortopathy, numerous investigators have undertaken studying different aspects of the disease. These efforts have successfully elucidated critical mechanisms and factors influencing disease development and progression in bicuspid aortopathy. To an extent, the motivation for this work has been the objective of defining a uniform, safe, and evidence-based set of guidelines for the medical and surgical management of these patients. Despite key advances, more research is needed. To this end, our group and others have focused on discovering and identifying novel, non-invasive histopathologic, and hemodynamic biomarkers which could potentially play a key role in further improving the care of patients with bicuspid aortopathy. By leveraging basic and translational research techniques, novel imaging modalities, and perhaps systemic biomarkers, improved risk prediction may result in more individualized treatment options and optimal management strategies.

## Author contributions

All authors listed have made a substantial, direct and intellectual contribution to the work, and approved it for publication.

### Conflict of interest statement

The authors declare that the research was conducted in the absence of any commercial or financial relationships that could be construed as a potential conflict of interest.
